# Eating Behaviours, Oral Hygiene, and Caries in a Population of Spanish Children with Divorced Parents: A Cross-Sectional Study

**DOI:** 10.3390/jcm12196189

**Published:** 2023-09-25

**Authors:** María Moya-López, Rafael Gómez-De Diego, María Carrillo-Díaz, Martín Romero-Maroto, Ana Ruiz-Guillén

**Affiliations:** 1 Department of Paediatric Dentistry, Rey Juan Carlos University, 28922 Alcorcón, Spain; maria.moya@urjc.es (M.M.-L.); maria.carrillo@urjc.es (M.C.-D.); martin.romero@urjc.es (M.R.-M.); ana.guillen@urjc.es (A.R.-G.); 2 Department of Prosthodontics, Rey Juan Carlos University, 28922 Alcorcón, Spain

**Keywords:** divorce, oral hygiene, dental caries, dietary habits, cross-sectional study

## Abstract

Dental caries are a public health problem that is influenced by dietary habits. This cross-sectional study aimed to investigate the feeding and hygiene habits that divorced parents exercise over their children compared to non-divorced parents, and how this may influence the rate of caries in their children. The data of participants (*n* = 174) with an average age of 12.17 ± 2.04 years were examined to assess the mean decayed/missing/filled teeth (DMFT) index, and they were asked questions about their oral hygiene habits. At the same time, their parents answered the parental feeding style questionnaire. A moderation analysis was conducted with family control of oral hygiene habit levels as an independent variable, decayed teeth as a dependent variable, and feeding control as a moderating variable. Results showed that divorced parents were found to have more problems in controlling their children’s hygiene and dietary habits, have less control over their children’s feeding, and make more use of instrumental feeding, which led to children of divorced parents having more caries. Despite the limitations linked to the cross-sectional design of the study and considering both the convenience sample and the impossibility of controlling for all aetiological factors linked to the development of caries, it can be concluded that children of divorced parents have an increased risk of tooth decay. However, parental controlled feeding interferes with the effect of family controlled oral hygiene habits on the decayed tooth, decreasing the rate of caries.

## 1. Introduction

The World Health Organization (WHO) defines oral health as an overall state that is free of orofacial pain, oral diseases, tooth loss, and limiting capabilities; oral health encompasses psychosocial well-being [1]. High global incidence rates of caries have forced health professionals to identify new factors to address this problem [2], as the formation of caries is generally preventable [3].

Dental caries has been shown to be strongly associated with dietary practices and the presence of bacteria in the oral environment [4]; therefore, sugar consumption remains an important factor in the development of this problem [5]. However, in recent years, family structure has begun to be considered as an influential factor in the development of caries [2]. The oral health knowledge, attitudes, and preventive practices of parents are directly related to the oral health of their children, as they are the primary caregivers [4,6]. In addition, the environment that parents create can influence children’s oral hygiene and eating habits [6]. On the basis that life changes and life dynamics can affect oral health habits, several authors have associated adverse childhood experiences, such as divorce, with poorer oral health [2,3,7]. Divorce is a specific transitional event that can be a period in which there is a greater likelihood of acquiring behavioural patterns that are risk factors for oral and general health [8,9]. Poor family functioning may be related to unhealthy eating behaviours in children [8].

Parents influence their children’s eating behaviour in a variety of ways, such as actively choosing the foods they eat, modelling food choices, creating food patterns, and using food practices to reinforce the eating behaviours that they consider appropriate [10]. However, these practices may be aimed at reducing stresses in children, such as those generated by an exogenous stressor such as divorce [2]. This is done via what is known as emotional feeding, in which food is used to calm or reduce children’s restlessness, and what is known as instrumental feeding, i.e., using food as a reward or punishment for their children [11,12]. These practices may arise due to the fact that the inter-parental conflicts generated by divorce leave parents emotionally exhausted and therefore less attentive to their children [13]. However, parents may also develop excessive or intrusive control strategies towards their children, exercising parental control as a strategy to manage, regulate, or control children’s behaviour [13].

To our knowledge, there are few studies that analyse the relationship between certain dietary habits and divorce. However, given the instability that divorce induces, it is expected to be a time of risk and tends to be associated with the presence of caries. Therefore, knowing the role of lifestyle-related risk indicators in the development of caries is the basis for the prevention of caries. The aim of this study was to examine the associations between risk behaviours for caries and family structure by using reports of feeding behaviours to collect information from participants and testing the hypothesis that parental limits on children’s dietary consumption may lead to a reduction in the prevalence of dental caries among school-aged children.

## 2. Materials and Methods

### 2.1. Settings and Participants

A population-based, observational, cross-sectional study was carried out at the Rey Juan Carlos University from December 2022 to April 2023. The sample was composed of 174 subjects (101 girls and 73 boys) with an average age of 12.17 ± 2.04 years, and one of the parents of each child; namely, the parent who accompanied the child to the check-up. This was a convenience sample, but an optimal sample size based on the results of the prevalence of caries in this area [14]; a prevalence of caries at 12 years of age in the permanent dentition of 30.1%. A random sample selection of 174 subjects was estimated to be representative of the cohort at that age based on three degrees of precision and with a confidence level of 95%. Data were collected via self-report questionnaires for each child and their parent attending the clinic for dental check-ups and by conducting oral examinations of the children. Before beginning the study, three examiners were trained and calibrated by a specialist. The result of the kappa statistics during the calibration process was 0.91 (good agreement). The clinical examinations were carried out by paediatric dentists, who used a flat-surface oral mirror, scanning probe, and compressed air.

All patients’ parents gave their informed consent for inclusion before they participated in the study; a researcher was available to answer questions about the survey. Children with any local or systemic conditions (medication, pathologies, and/or disabilities) that could affect their oral health status, candidates who did not provide informed consent, and candidates with a mother tongue other than Spanish were excluded from the study.

The study was conducted in accordance with the Declaration of Helsinki, and the protocol was approved by the Ethics Committee of the Rey Juan Carlos University (approval No. 1409202219622).

### 2.2. Measures

An analysis of the socio-demographic characteristics of the sample was carried out by dividing the sample according to families with divorced parents and families with non-divorced parents (Table 1). The sample was balanced in terms of sex and age. Mothers had a higher level of education in 53.4% of the sample; 70.1% of the participants were employed full time. Regarding the educational level of the fathers, 78.2% had higher or secondary education.

In addition, the following variables were taken into account:

*Oral health status:* The DMFT index (sum of decayed teeth (DT), missing teeth (MT), and filled teeth (FT)) is one of the most used indices in dental caries epidemiologic surveys. This diagnostic criterion, which was established by the World Health Organization [14], quantifies dental health status based on the number of carious, missing, and filled teeth.

*Use of dental floss*: Use of dental floss was determined via the children’s dichotomous response to a question to ascertain if they used dental floss (yes/no).

*Hygiene habits:* This variable was assessed by asking the children the question, “In general, how often do you brush your teeth?” The response format was a 5-point Likert-type scale, ranging from 1 (I do not usually brush my teeth) to 5 (3 times a day).

*Regular visits to the dentist:* Children were asked: “In general, how often do you go to the dentist?” The response format was a 5-point Likert-type scale, with a range from 1 (I have never gone) to 5 (every 6 months).

*Parental Feeding Style Questionnaire (PFSQ):* The Parental Feeding Style Questionnaire was used [15]. The response format was a 5-point Likert-type scale, with a response range from 1 (never) to 5 (always). This questionnaire was composed of four scales: emotional eating (5 items, e.g., I give him something to eat to make him feel better when he is angry); instrumental eating (4 items, e.g., I reward my child with something to eat when he behaves well); prompting and encouragement to eat (8 items, e.g., I praise my child if he eats what I give him); and control over eating (10 items, e.g., I decide how many snacks my child should have). The sum of parental responses was calculated to determine a final score on the questionnaire, high scores indicate that a greater tendency to adopt a specific style. Cronbach’s alpha reliability was 0.77 for this scale.

### 2.3. Statistical Analysis

The statistical program SPSS (Statistical Package of the Social Sciences Program for Windows 28.0) was used for statistical calculations. A χ^2^ test was used to assess the difference in toothbrushing, flossing, dental visits, and family situation problems with respect to maintaining healthy dental hygiene and eating habits among children of divorced and non-divorced parents. The Student’s *t*-test was used to investigate whether children of divorced parents had a different parental feeding style and poorer oral health. For the t of independent samples, Cohen’s d was calculated. According to Cohen [16], low Cohen’s d values are ≈0.2, medium ones are ≈0.5, and high ones are ≈0.8. Cohen [16] also considered small effect size values to be ≈0.01, medium ones to be ≈0.06, and those large enough to be considered to be ≈0.14. The Hayes PROCESS module (version 3.3) was used to perform multiple simple moderation analyses (Model 1) [17]. Cronbach’s alpha was also obtained to evaluate the internal consistency of the instruments. The results are expressed as means ± standard deviation, and percentages and differences were considered significant at *p* level < 0.05.

## 3. Results

### 3.1. Pearson Correlations between Study Variables

Pearson correlations between study variables are presented in Table 2. Mother’s employment was positively associated with control over eating (r^2^ = 0.155, *p* ≤ 0.05) and inversely associated with instrumental feeding (r^2^ = −0.153, *p* ≤ 0.05). Hygienic dental habits were positively associated with control over eating (r^2^ = 0.168, *p* ≤ 0.05) and inversely associated with instrumental eating (r^2^ = −0.235, *p* ≤ 0.01). Instrumental eating was inversely associated with socioeconomic level (r^2^ = −0.266, *p* ≤ 0.01). Decayed teeth were inversely associated with control over eating (r^2^ = −0.200, *p* ≤ 0.01) and missing teeth was inversely associated with father’s employment (r^2^ = −0.167, *p* ≤ 0.05) (Table 2).

### 3.2. Comparative Analysis of Oral Care in Children of Divorced and Non-Divorced Parents

A comparative analysis was carried out between families with divorced and non-divorced parents with regard to their children’s hygiene habits. It was found that children of divorced parents brushed their teeth significantly less (χ^2^ = 10.578 *); no differences were found regarding flossing (χ^2^ = 3.551). Differences in dental visits were found, with children of divorced parents visiting the dentist less frequently (χ^2^ = 11.988 *). As to whether the family situation impeded hygiene habits, it was found that families with divorced parents had more problems in the control of hygiene habits (χ^2^ = 14.966 **); the same was true for the control of dietary habits (χ^2^ = 8.001 *) (Table 3).

### 3.3. Comparative Analysis of the Parental Feeding Style Questionnaire (PFSQ) and Oral Health in Children of Divorced and Non-Divorced Parents

Significant differences were found in terms of dietary control. Divorced parents had less control over their children’s feeding (*p* = 0.018). However, in terms of instrumental feeding, divorced parents were found to use this technique more (*p* = 0.047) with their children. In terms of food promotion and encouragement, no significant differences were found (*p* = 0.731). In addition, the children of divorced parents had more caries than the children of non-divorced parents (*p* = 0.013). All differences were found with small effect sizes (Table 4).

### 3.4. Analysis of the Moderation of Family Hygiene Control Habits on Feeding Control and Decayed Teeth in Children with Divorced Parents

A moderation analysis was conducted with the level of family control hygiene habits as an independent variable, decayed teeth as a dependent variable, and feeding control as a moderating variable. The regression analysis, in which the level of family control hygiene habits was considered a predictor of decayed teeth, had *p* = 0.001; the same occurred for the moderator variable (*p* = 0.01). However, a significant value was obtained for the interaction between the independent and moderator variable (*p* = 0.001; SE = 0.02; [−0.14, −0.05]). Obtaining a significant value for this interaction indicates the presence of a moderator effect, which suggests that control feeding interferes with the effect of parental control hygiene habits on decayed teeth.

**Figure 1:**
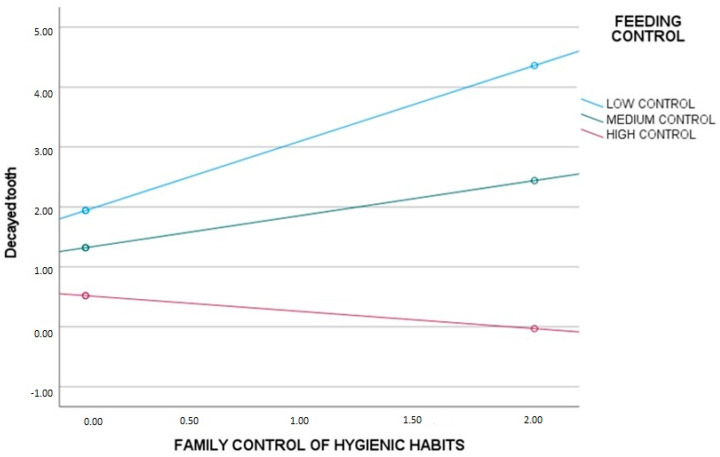
Analysis of the moderation of family hygiene control habits on feeding control and decayed teeth in children with divorced parents.

To determine when control feeding has a moderating effect in the main model, the significance level and the upper and lower limits were analysed. In this case, it was noted that control feeding has a moderating effect only in those with medium control feeding levels (*p* = 0.01; SE = 0.20; [0.18, 0.99]) and even more so in those with low control feeding levels (*p* = 0.0001; SE = 0.25; [0.76, 1.76]). However, it has no effect on those with high control feeding levels (*p* = 0.35; SE = 0.31; [−0.90, −0.33]) (Figure 1). In summary, it could be observed that parental control feeding interferes with the effect of family control hygiene habits on the decayed teeth.

## 4. Discussion

The results of this study suggest that the hygiene and feeding practices of divorced parents, compared to those of non-divorced parents, may influence the oral health of their children, leading to a higher prevalence of caries; the family imposes the most important effects on the psychological, physical, and social aspects of health from the time the child is born [18].

According to previous studies [3,7], a high proportion of children who had suffered adverse childhood experiences, such as divorce, had dental problems compared to children who had not suffered adverse childhood experiences. Along these lines, a study by Bright et al. [3] argued that social factors linking adverse experiences to poor oral health could include family routines and functioning, as well as parental attitudes towards oral health. Levin and Currie [19] also linked the family environment to oral health, finding that promoting oral health can be achieved by creating a mealtime routine and developing a positive and open family relationship. Mauskopf et al. [8] also found that children in families with divorced parents consumed more sugary drinks than children with non-divorced parents, finding that a higher frequency of family routines protected against the consumption of these drinks; this is something to be taken into account, as caries are strongly associated with dietary factors [5]. However, in a study conducted by Folayan et al. [20], contrary to the findings of the present study, no relationship was found between adverse childhood experiences and caries or poorer oral hygiene, although this study was conducted with an older sample compared to the present study.

The findings of this study are consistent with those of previous studies, such as the one conducted by Mattila et al. [7], which found that there was a higher prevalence of tooth decay in families with complicated relationships, and that the rate of tooth decay was higher in children whose parents used sweets to calm their children’s temper tantrums. Instrumental feeding has been linked to an increased likelihood of children consuming ultra-processed food, sugary drinks, snacks, and pastries [11], which may promote tooth decay. Mothers who use instrumental feeding practices offer their children energy-dense, nutrient-poor foods such as sweets, biscuits, and chocolate [21].

Vollmer and Baietto [22] found that when there is less parental control over eating, children have fewer preferences for healthy foods such as fruit, and they concluded that regulating children’s emotions with food, using food as a reward, and restricting food for health reasons are positively associated with children’s preferences for high-fat and high-sugar foods.

The results of the present study showed that as soon as parental control of food intake is introduced, there is a decrease in the rate of caries; this coincides with the results of the study by Bonotto et al. [5], in which it was found that there was a lower rate of caries in children whose parents reduced dietary fat intake. It was also found that concern for controlling unhealthy foods is part of a behavioural pattern that includes different healthy eating measures, such as a decrease in sugar consumption, indicating that setting limits on snacking is a protective factor against caries, an association that was maintained even after controlling for variables such as brushing frequency and the presence of dental plaque. Other studies have also found that an authoritative parenting style, i.e., parents exercising control over their children, can be effective in reducing the consumption of “empty” calories in early childhood [23].

However, studies such as one by Brown and Ogden [24] have found that when parents exert control over their children’s diets, children eat more of both healthy and unhealthy foods. These authors concluded that using food to change behaviour may further disconnect food from its hunger-satisfying function and foster a more problematic relationship with food. In line with these results, Nembhwani and Winnier [12] also found that imposing a type of diet that children do not like may result in a preoccupation with less healthy food that contributes to increased caries, as it was found that when there was greater parental control of the diet, the rate of caries increased. However, when there was instrumental feeding, the rate of caries decreased, which is the opposite of the results found in the present study.

To our knowledge, no previous studies have been found that analyse dental hygiene in the children of divorced parents, but it has been found that a close relationship with at least one parent and perceived fair parenting by the children are associated with better dental hygiene [19]. However, the relationship between family structure and toothbrushing may disappear when family relationships and mealtime routines are taken into account.

### Strengths and Limitations

The main strength of this study is the focus on an understudied Spanish population, and the results have important practices repercussions in terms of deepening the prevention of caries. In this way, caries improvement programmes could be implemented in primary care centres or in dental clinics during the child’s first dental visit with his or her parents, such as the PROTECT project in Italy, which aims to promote the dissemination of information on the prevention and treatment of head and neck diseases [25], as well as the FRAMM Guideline in Sweden [26].

As tooth decay continues to be a serious public health problem [18], future research should explore the relationship between oral health and family dynamics, as the results of the present study show that the influence of negative interparental relationships results in a higher prevalence of caries in their children. It would also be interesting to carry out statistical correlation and regression analyses for stronger conclusions.

This study has certain limitations. Firstly, factors associated with the prevalence of dental caries were analysed with a cross-sectional design, which does not allow causality to be established. Caries is a chronic condition involving the interaction of different factors over time, and longitudinal studies are more suitable for investigating the role of dietary habits in the development of dental caries in children. In addition, self-reports of dietary intake can be biased by social desirability [27]. Another possible limitation is that no record was kept of what the children ate, as well as other aetiological factors of caries such as fluoride use. Finally, the sample that was used was a convenience sample, which was obtained from a specific segment of the population in the community of Madrid, potentially limiting the possibility of generalising the results.

## 5. Conclusions

The present study showed an association between the presence of caries in the children of divorced parents and how feeding habits influence this association. Divorced parents had less control over their children’s hygiene and feeding, used more instrumental feeding techniques, and had children with a higher prevalence of caries compared to the children of non-divorced parents. However, as divorced parents exercised more control over feeding, the rate of caries in their children decreased.

## Figures and Tables

**Table 1:** Demographic characteristics of participants.

Variables	Non-Divorced	Divorced	N (%)/M(DS)
**Sex**			
Male	39	34	73 (41.4%)
Female	48	53	101 (58.6%)
Total N (%)	87 (50%)	87 (50%)	174 (100%)
**Age of children**			
M (DS)	11.83 (1.74)	12.50 (2.27)	12.17 (2.04)
**Mother’s educational level**			
No studies	1	8	9 (5.1%)
Primary school	13	9	22 (12.5%)
Secondary	24	26	50 (28.7%)
Higher education	49	44	93 (53.4%)
Total N (%)	87 (50%)	87 (50%)	174 (100%)
**Father’s educational level**			
Primary school	24	14	38 (21.8%)
Secondary	28	40	68 (39.1%)
Higher education	35	33	68 (39.1%)
Total N (%)	87 (50%)	87 (50%)	174 (100%)
**Mother’s employment**			
Employed part time	14	21	35 (20.1%)
Employed full time	65	57	122 (70.1%)
Unemployed	8	9	17 (9.8%)
Total N (%)	87 (50%)	87 (50%)	174 (100%)
**Father’s employment**			
Employed part time	0	7	7 (4.02%)
Employed full time	82	76	158 (90.8%)
Unemployed	5	5	10 (5.74%)
Total N (%)	87 (50%)	87 (50%)	174 (100%)

**Table 2:** Pearson Correlations between Study Variables (n = 174).

	2	3	4	5	6	7	8	9	10
1. Socioeconomic Level	0.213 **	0.02	0.022	0.024	−0.095	−0.266 **	0.032	−0.129	−0.051
2. Mother’s employment		−0.127	0.074	0.155 *	0.075	−0.153 *	−0.025	0.015	−0.072
3. Father’s employment			0.07	0.036	−0.013	0.05	−0.026	−0.058	−0.167 *
4. Hygienic dental habits				0.168 *	0.044	−0.235 **	−0.063	0.038	−0.042
5. Control over Eating					0.178*	−0.023	−0.200 **	0.001	−0.072
6. Promoting and Encouragement to eat						0.129	0.012	0.076	0.117
7. Instrumental Eating							0.133	0.072	0.027
8. Decayed teeth								−0.098	0.289 **
9. Filled Teeth									0.065
10. Missing Teeth									

Note: * Correlation is significant at the 0.05 level; ** Correlation is significant at the 0.01 level.

**Table 3:** Comparative analysis of oral care in children of divorced and non-divorced parents.

Variables	Children of Non-Divorced Parents N	Children of Divorced Parents N	χ^2^ (Sig.)
**Children’s toothbrushing**			
Less than once a day	1	7	10.578 *
Once a day	22	30	
Twice a day	43	41	
Three times a day	21	9	
N (%)	87	87	
**Flossing of the child’s teeth**			
No	69	78	3.551
Yes	18	9	
N (%)	87	87	
**Children’s dental visits**			
I have never been	1	3	11.098 *
I have gone only when I have a problem or pain	10	21	
I go every 2–3 years	1	5	
I go once a year	12	14	
I go every 6 months	63	44	
N (%)	87	87	
**Family situation impedes dietary habits**			
No, not at all	74	66	8.001 *
A little	12	11	
Quite a lot	1	7	
N (%)	0	3	
**Family situation impedes hygienic dental habits**			
No, not at all	82	65	14.966 **
A little	4	8	
Quite a lot	1	5	
N (%)	0	9	

Note: * Correlation is significant at the 0.05 level; ** Correlation is significant at the 0.01 level.

**Table 4:** Comparative analysis of the parental feeding style questionnaire (PFSQ) and oral health in children of divorced and non-divorced parents.

	Non-Divorced Parents M (DS)	Divorced M (DS)	*p*-Value	D Cohen
PFS QUESTIONNAIRE				
Control	33.96 (6.73)	31.45 (7.08)	0.018	0.36
Instrumental feeding	5.63 (2.06)	6.42 (3.06)	0.047	0.30
Prompting and encouragement to eat	32.09 (6.14)	32.40 (5.75)	0.731	0.05
ORAL HEALTH				
Decayed	0.85 (1.92)	1.68 (2.44)	0.013	0.38
Missing	0.06 (0.33)	0.09 (0.42)	0.690	0.06
Filled	1.48 (2.13)	1.98 (2.37)	0.142	0.22

## Data Availability

Data will be made available on request.
